# New Challenges in Wood and Wood-Based Materials II

**DOI:** 10.3390/polym15061409

**Published:** 2023-03-12

**Authors:** Lubos Kristak, Roman Réh, Ivan Kubovský

**Affiliations:** Faculty of Wood Sciences and Technology, Technical University in Zvolen, 96001 Zvolen, Slovakia

Wood is a natural material that is available in large quantities and is easy to produce, making it the perfect material to consider for the circular economy. Its importance has dramatically increased in recent years. This increase is accompanied by the development of new research methods that open new possibilities in the areas related to wood and wood products in the process of their production, processing, and final use. The main topics of this Special Issue were knowledge of the quality of wood and other lignocellulose materials; the processes of their effective utilization and processing for more efficient processing; the adoption of techniques and research around using wood for environmentally friendly composite production and the positive impact of this on the environment; wood’s interaction with solid substances and with different mechanical loads, chemicals and other substances; and the different forms of energy and surface modification of wood and wood composites.

Section 1 *Wood-Based Panels*

Over the last 50 years, the use of wood adhesives in the manufacturing of wood-based panel goods has increased the efficiency of wood resources. Wood adhesives are becoming more popular as the need for wood-based panels grows [1]. Traditional wood-based panels are produced with synthetic, formaldehyde-based adhesives that are commonly made from fossil-derived constituents, such as urea, phenol, melamine, etc. Along with their numerous advantages, such as chemical versatility, high reactivity, and excellent adhesive performance, these adhesives are characterized by certain problems, and connected with hazardous volatile organic compounds (VOCs) [2,3]. Recently, increased amounts of biobased adhesives have been used in the production of wood composites to meet the current need for the development of sustainable and innovative materials which will make the wood-based panel industry more sustainable and lower its dependence on fossil fuels [4]. Increased interest in developing sustainable adhesives from different renewable biomass feedstock focuses mostly on lignin, tannin, starch, and proteins [5]. 

Authors Jorda et al. [6] focused their research on bio-based adhesive formulation consisting of Quebracho tannin extract and furfural. They investigated its usability as an adhesive for five-layered beech (Fagus sylvatica L.) plywood, detailing its press parameters and their influence on its physical and mechanical properties. The prepared fully bio-based adhesive formulation showed good viscosity and curing behavior at a relatively low temperature (100 °C), good bending, and acceptable tensile shear strength in a dry environment, which was comparable to industrial applicated PF adhesive. The authors have concluded that the proposed Quebracho tannin and furfural formulation can improve and contribute to recyclability for specific interior plywood applications as a key element of the bio-based circular economy. Another piece of research conducted by Jorda et al. [7] focused on using adhesive systems for flax fiber-reinforced beech plywood. Epoxy resin, urea-formaldehyde, melamine-urea formaldehyde, isocyanate MDI prepolymer, and polyurethane had a different role in improving its mechanical properties. Epoxy resin showed excellent properties in the case of flax fiber reinforcement, while urea-formaldehyde, melamine urea-formaldehyde, and isocyanate MDI prepolymer helped improve the modulus of elasticity, modulus of rupture, shear strength, and screw withdrawal resistance. On the other hand, the tensile strength was lowered in the case of these adhesive systems. Polyurethane was the last tested adhesive system that showed the lowered mechanical properties of flax fiber-reinforced plywood. As the authors concluded, further research is mandatory to determine the influence of press parameters, in addition to factors such as veneer and flax fiber manufacturing, and pre-treatment of the flax fiber [8,9]. Research in the area of fiber reinforcement used for veneer-based products has become recently very popular. Alongside flax fibers, several natural fibers such as hemp or kenaf, or synthetic fibers such as carbon have shown very good potential in increasing the mechanical properties of veneer-based products [10,11,12].

Alongside plywood, particleboards are another major wood-based product in global trade. Their global popularity and production have seen an upward trend in recent years [13,14,15]. In terms of quality assessment, particleboards have only a few disadvantages, and flammability is among them [16]. Several researchers have evaluated the flammability of wood-based composite materials [17,18,19]. The assumption of a fire hazard requires an appropriate description of the fire ignition and fire development [20,21]. The authors Turekova et al. [22] focused in their research on the significance of the influence of heat flux density and particleboards’ properties on their thermal resistance (time to ignition). The obtained results showed that the time to ignition depended significantly on the thickness of the particleboard. Additionally, the linear dependence of the distance of the sample from the heat source was confirmed.

Glued-laminated timber (Glulam) is a wood-based product preferred for wide-span timber structures due to its lower variability in strength properties, and the possibility of producing it in almost any length and shape [23]. Hollow glulam beam has some advantages compared to traditional solid glulam beams, such as the convenience for wiring construction, comparably lightweight, and higher strength-to-weight ratio [24]. Perkovic et al. in their paper [25] presented innovative hollow Glulam elements intended for log-house construction. The research also involved testing the compression strength of elliptical hollow cross-section Glulam specimens made of softwood and hardwood, as well as full cross-section glue-laminated softwood timber specimens. The results showed that the compression strength perpendicular to the grain of hollow specimens decreased by about 55% compared to the full cross-section. By removing the holes in the central part of the cross-section, the stress was reduced. Additionally, the distance of the holes from the edges defined the local cracking. This research followed the long-term research focused on innovative hollow Glulam elements [26,27].

Cross-laminated timber (CLT) as a structural, plate-like timber product gained global recognition in the last three decades. The cross-laminated configuration improves the stability, rigidity, and mechanical properties of the product [28]. CLT is commonly utilized as a wall, roof, floor diaphragm, and other structural components. The connections between structural components (wall to wall, wall to floor, floor to floor, wall to foundation, and wall to concrete cores) must be designed for adequate strength, stiffness, and ductility when CLT panels are used in structures [29,30]. Research conducted by Abdoli et al. [31] was focused on the withdrawal resistance of nine types of conventional fasteners (stainless steel nails, concrete nails and screws, drywall screws, three types of partially and fully threaded wood screws, and two types of lag screws) with three loading directions and two-layer arrangements in 3-ply CLTs made of poplar and fir. Research results confirmed that the fastener type had the most significant impact on the withdrawal resistance, so changing the fastener type from nails to screws increased it by about 5–11 times, which is in accordance with other studies.

Wood frame walls have also gained popularity in recent decades. The frames are usually made from spruce or pine timber surrounded (mostly) by OSB casing and filled with thermal insulation material [32,33]. Szcepanski et al. [34] tested three walls of different widths, with and without two different sizes of openings, in several locations, to monitor the change in their natural frequencies under dynamic loads. The results showed that the effect of the size and location of the openings on the natural frequency is significant (and more sensitive for the location than for the sizes). The relative change in the natural frequencies of a wall without and with an opening in a specific place could be up to 30%. The authors concluded that the appropriate size and place were found to be small openings at the top of the walls.

Section 2 *Wood*

Wood is a valuable industrial and building material with many excellent properties in its natural state. Modification of wood is applied to overcome weak points that are mainly related to hygroscopicity, low dimensional stability, hardness, wear resistance, low resistance to different forms of radiation (UV, IR, etc.), and low resistance to bio-deterioration against fungi and termites [35,36,37]. Though many aspects of wood modification treatments are known, the fundamental influence of the process on product performance, the environment, and end-of-life scenarios remains relatively unknown [38,39]. A very popular and ecological method is the thermal modification of wood. Thermal modification is a well-established commercial technology for improving the durability and dimensional stability of wood [40,41,42]. 

The unique mechanical and acoustical properties of wood and its aesthetic appeal still make it the material of choice for musical instruments. The desire of musical instrument manufacturers to reduce the negative characteristics of raw material wood through special modification processes has existed for a century now [43,44]. In research by Danihelova et al. [45], the authors investigated the suitability of thermally modified (ThermoWood process) Norway spruce and sycamore maple for special wood products, mainly for musical instruments. Selected physical and acoustical characteristics (PACHs) including density, dynamic modulus of elasticity along the wood grain, specific modulus, speed of sound along the wood grain, resonant frequency, acoustic constant, logarithmic decrement, loss coefficient, acoustic conversion efficiency, sound quality factor and the timbre of sound were evaluated. Fast Fourier transform was used to analyze the sound produced. Based on the results, the mild thermal modification resulted in a decrease in mass and density, and in an increase in the speed of sound and dynamic modulus of elasticity at all temperatures of modification. The thermally modified wood showed higher sound radiation and lower loss coefficients than unmodified wood, and influenced the timbre of sound of both wood species.

Wood is one of the most sustainable, aesthetically pleasing, and environmentally friendly materials; however, the hazards of fire make wood a very desirable material for further investigation. As well as having ignition resistance and a low heat release rate, wood products are required to resist burn-through and maintain structural integrity when exposed to fire or heat. The changes in the residential fire environment, and the lack of fire behavior training are significant factors that are contributing to the continued climb in traumatic deaths and injuries to firefighters [46,47,48,49,50]. In research by Gaff et al. [51], the authors investigated the effect of synthetic and natural flame retardants on the flammability characteristics and chemical changes in thermally modified meranti wood. The basic chemical composition was evaluated to clarify the relationships between temperature modifications and incineration. Weight loss, burning speed, the maximum burning rate, and the time taken to reach the maximum burning rate were evaluated. The thermal modification did not confirm a positive contribution to the flammability and combustion properties of meranti wood. The effect of synthetic retardant on all combustion properties was significantly higher compared to that of natural retardant.

Some significant factors affecting the behavior of wood during exposure to fire are its chemical composition, wood species, density, moisture content, permeability, anatomy, and aging process [52,53,54]. Authors Zachar et al. [55] provided research dealing with the effect of the age of oak wood (0,10,40,80, and 120 years) on its fire resistance. The authors determined the chemical composition of wood by the wet chemistry method, and elementary analysis was performed according to ISO standards. From the fire’s technical properties, the flame ignition, spontaneous ignition temperature, and mass burning rate were evaluated. Results showed that lignin content did not change, the content of extractives and cellulose increased and the content of holocellulose decreased with the higher age of the wood. The elementary analysis confirmed the lowest proportional content of nitrogen, sulfur, and phosphor, and the highest content of carbon in the oldest wood. The difference among the values of spontaneous ignition activation energy is clear evidence of higher resistance to the initiation of older wood in comparison with younger wood. The research confirmed that the oldest sample is the least thermally resistant, due to the different chemical compositions.

Steam treatment is often applied to wood to improve the stability and permeability of the wood, obtain a desirable color and soften the wood [56,57,58]. In the paper by authors Dudiak et al. [59], the differences in the color changes of unsteamed and steamed beech wood were caused by long-term exposure to sunlight on the surface of the wood in interiors for 36 months. The lower value of the total color difference of steamed beech wood indicated the fact that steaming of beech wood with saturated water steam had a positive effect on the color stability and partial resistance of steamed beech wood to the initiation of photochemical reactions induced by UV–VIS wavelengths of solar radiation. Spectra ATR-FTIR analyses declared the influence of UV–VIS components of solar radiation on unsteamed and steamed beech wood, and confirmed the higher color stability of the steamed beech wood. Another study, conducted by Dzurenda et al. [60], dealt with maple wood steamed with a saturated steam-air mixture to give a pale pink-brown, pale brown, and brown-red color. Subsequently, samples of unsteamed and steamed maple wood were irradiated with a UV lamp in a Xenotest after drying to test the color stability of the steamed maple wood. The results showed that the surface of unsteamed maple wood changes color far more markedly under the influence of UV radiation than the surface of steamed maple wood. Differential ATR-FRIT spectra confirmed the effect of UV radiation on unsteamed and steamed maple wood and confirmed the higher color stability of the steamed maple wood.

The protection of wood is particularly demanding in adverse environments. The use of wood in marine environments is a major challenge due to the high sensitivity of wood to both water and marine microorganisms [61,62]. Another interesting study by Filgueira et al. [63] focused on the development of a new green methodology based on the laccase-assisted grafting of lauryl gallate onto wood veneers, to improve their marine antifouling properties. Different wood species (beech, pine, and eucalyptus) were effectively hydrophobized through the enzymatic treatment. The reaction condition played an important role in the extent of hydrophobization; the treated wood species were also a major factor. The results observed in the study confirmed the potential efficiency of laccase-assisted treatments to improve the marine antifouling properties of wood.

With increasing energy demand and requirements for environmental conservation, the replacement of petroleum-based materials with bio-based materials is an interesting opportunity. Transparent wood is an example of a multifunctional wood composite. Optically transparent wood integrates mechanical and optical properties, and is a promising contender for intelligent buildings, structural optics, and photonics applications [64,65,66,67,68]. In research conducted by Wachter et al. [69], the effect of monochromatic UV-C radiation on transparent wood was evaluated. Samples of basswood were treated using a lignin modification method to preserve most of the lignin, and subsequently impregnated with refractive-index-matched types of acrylic polymers. Optical and mechanical properties were measured to describe the degradation process over 35 days. Results confirmed that exposure to UV-C radiation has a significant effect on the color of transparent wood. Samples became darker with increasing exposure time, and their color shifted towards shades of red and yellow; this can be possibly explained by the reactivation of chromophores. Additionally, the transmittance of light was significantly affected by UV-C radiation. The influence of UV-C radiation on hardness was significantly lower than in the case of optical properties. The measured values showed that the resulting hardness of transparent wood depends mainly on the hardness of the acrylic polymer used. 

We would like to thank our Section Managing Editor, Chris Chen for his professional attitude and assistance with publishing.
